# Supplementary material for: The Impact of the COVID-19 Pandemic on Hospital Services for Patients with Cardiac Diseases: A Scoping Review
Source: Int J Environ Res Public Health. 2022 Mar 8;19(6):3172. doi: 10.3390/ijerph19063172 (PMC8953098; doi:10.3390/ijerph19063172)
Supplement: Supplementary file 1 [file ijerph-19-03172-s001.zip › ijerph-1596722-supplementary.pdf]

Supplementary material S1 - Preferred Reporting Items for Systematic reviews and Meta-Analyses extension for Scoping Reviews (PRISMA-ScR) Checklist

SECTION	ITEM	PRISMA-ScR CHECKLIST ITEM	REPORTED ON PAGE #
**TITLE**			
Title	1	Identify the report as a scoping review.	Title Page
**ABSTRACT**			
Structured summary	2	Provide a structured summary that includes (as applicable): background, objectives, eligibility criteria, sources of evidence, charting methods, results, and conclusions that relate to the review questions and objectives.	2
**INTRODUCTION**			
Rationale	3	Describe the rationale for the review in the context of what is already known. Explain why the review questions/objectives lend themselves to a scoping review approach.	3
Objectives	4	Provide an explicit statement of the questions and objectives being addressed with reference to their key elements (e.g., population or participants, concepts, and context) or other relevant key elements used to conceptualize the review questions and/or objectives.	3
**METHODS**			
Protocol and registration	5	Indicate whether a review protocol exists; state if and where it can be accessed (e.g., a Web address); and if available, provide registration information, including the registration number.	
Eligibility criteria	6	Specify characteristics of the sources of evidence used as eligibility criteria (e.g., years considered, language, and publication status), and provide a rationale.	5
Information sources*	7	Describe all information sources in the search (e.g., databases with dates of coverage and contact with authors to identify additional sources), as well as the date the most recent search was executed.	4
Search	8	Present the full electronic search strategy for at least 1 database, including any limits used, such that it could be repeated.	4
Selection of sources of evidence†	9	State the process for selecting sources of evidence (i.e., screening and eligibility) included in the scoping review.	6
Data charting process‡	10	Describe the methods of charting data from the included sources of evidence (e.g., calibrated forms or forms that have been tested by the team before their use, and whether data charting was done independently or in duplicate) and any processes for obtaining and confirming data from investigators.	6
Data items	11	List and define all variables for which data were sought and any assumptions and simplifications made.	6
Critical appraisal of individual sources of evidence§	12	If done, provide a rationale for conducting a critical appraisal of included sources of evidence; describe the methods used and how this information was used in any data synthesis (if appropriate).	Not applicable
Synthesis of results	13	Describe the methods of handling and summarizing the data that were charted.	7

SECTION	ITEM	PRISMA-ScR CHECKLIST ITEM	REPORTED ON PAGE #
**RESULTS**			
Selection of sources of evidence	14	Give numbers of sources of evidence screened, assessed for eligibility, and included in the review, with reasons for exclusions at each stage, ideally using a flow diagram.	8
Characteristics of sources of evidence	15	For each source of evidence, present characteristics for which data were charted and provide the citations.	8
Critical appraisal within sources of evidence	16	If done, present data on critical appraisal of included sources of evidence (see item 12).	Not applicable
Results of individual sources of evidence	17	For each included source of evidence, present the relevant data that were charted that relate to the review questions and objectives.	8-12
Synthesis of results	18	Summarize and/or present the charting results as they relate to the review questions and objectives.	8-12
**DISCUSSION**			
Summary of evidence	19	Summarize the main results (including an overview of concepts, themes, and types of evidence available), link to the review questions and objectives, and consider the relevance to key groups.	13
Limitations	20	Discuss the limitations of the scoping review process.	15
Conclusions	21	Provide a general interpretation of the results with respect to the review questions and objectives, as well as potential implications and/or next steps.	14-15
**FUNDING**			
Funding	22	Describe sources of funding for the included sources of evidence, as well as sources of funding for the scoping review. Describe the role of the funders of the scoping review.	17

JBI = Joanna Briggs Institute; PRISMA-ScR = Preferred Reporting Items for Systematic reviews and Meta-Analyses extension for Scoping Reviews.

* Where *sources of evidence* (see second footnote) are compiled from, such as bibliographic databases, social media platforms, and Web sites.

† A more inclusive/heterogeneous term used to account for the different types of evidence or data sources (e.g., quantitative and/or qualitative research, expert opinion, and policy documents) that may be eligible in a scoping review as opposed to only studies. This is not to be confused with *information sources* (see first footnote).

‡ The frameworks by Arksey and O'Malley (6) and Levac and colleagues (7) and the JBI guidance (4, 5) refer to the process of data extraction in a scoping review as data charting.

§ The process of systematically examining research evidence to assess its validity, results, and relevance before using it to inform a decision. This term is used for items 12 and 19 instead of "risk of bias" (which is more applicable to systematic reviews of interventions) to include and acknowledge the various sources of evidence that may be used in a scoping review (e.g., quantitative and/or qualitative research, expert opinion, and policy document).

From: Tricco AC, Lillie E, Zarin W, O'Brien KK, Colquhoun H, Levac D, et al. PRISMA Extension for Scoping Reviews (PRISMAScR): Checklist and Explanation. Ann Intern Med. 2018;169:467–473. ***doi: 10.7326/M18-0850***.

Supplementary material S2 – Search strategy

Database searched	via	Years of coverage	Records	Records after duplicates removed
Embase	Embase.com	1971 - Present	4282	4206
Medline ALL	Ovid	1946 - Present	4429	2071
**Total**			**8711**	**6277**

Embase.com 4282

(pandemic/mj/exp OR 'natural disaster'/mj/exp OR disaster/mj/exp OR hurricane/mj/exp OR earthquake/mj/de OR 'coronavirus disease 2019'/mj/de OR 'Severe acute respiratory syndrome coronavirus 2'/mj/de OR (pandemic* OR disaster* OR hurricane* OR flood OR flooding OR earthquake* OR coronavirus-disease-2019 OR covid19 OR covid-19 OR sars-cov-2 OR 2019-novel-coronavirus OR 2019-ncov OR lockdown OR lock-down OR typhoon* OR (natural NEAR/3 hazard*)):ti) AND ('non communicable disease'/de OR neoplasm/exp OR 'malignant neoplasm'/de OR 'cancer patient'/exp OR 'heart disease'/exp OR cardiology/de OR 'neurologic disease'/de OR 'cancer screening'/de OR 'cancer surgery'/de OR (('primary medical care'/exp OR 'general practitioner'/de OR 'general practice'/de OR 'family medicine'/de OR 'emergency ward'/exp OR 'emergency care'/de) AND ('health care utilization'/exp OR consultation/de)) OR 'diabetes mellitus'/exp OR 'rheumatic disease'/exp OR 'mental health service'/de OR 'cardiovascular disease'/exp OR 'cerebrovascular disease'/exp OR 'chronic kidney failure'/exp OR 'obstructive airway disease'/exp OR 'chronic respiratory tract disease'/de OR 'cerebrovascular accident'/exp OR hypertension/exp OR 'chronic disease'/de OR 'neurologic disease'/exp OR (((non-communicab* OR noncommunicab*) NEAR/3 disease) OR cancer OR ((heart OR cardiovascul* OR cerebrovascul*) NEAR/3 (disease* OR patient* OR emergenc*)) OR cardiolog* OR neurolog* OR oncolog* OR ((((emergency OR acute) NEAR/3 (ward* OR care OR department*)) OR (general NEXT/1 (practi*)) OR ((primary OR family) NEXT/2 (healthcare* OR care OR doctor*))) AND (utilization* OR utilisation* OR delay* OR time OR visit* OR consultation* OR barrier* OR access* OR challenge* OR vulnerab* OR attendan* OR hesita*)) OR diabet* OR rheumat* OR (mental NEAR/3 (health-care* OR health-service* OR healthcare*)) OR (chronic NEAR/3 (kidney OR renal OR respirator* OR lung* OR pulmonar*) NEAR/3 (failure OR disease*)) OR asthma* OR bronchitis OR ((lung* OR pulmonar*) NEAR/3 (emphysem*)) OR ((cerebrovascul* OR cerebro-vascul*) NEAR/3 accident*) OR stroke OR hypertens* OR chronic-disease* OR (surg* NEAR/3 emergenc*)):ab,ti) AND ('disease exacerbation'/mj/de OR 'recurrent disease'/mj/de OR 'avoidance behavior'/mj/de OR 'recurrence risk'/mj/de OR 'undiagnosed disease'/mj/de OR 'diagnostic error'/mj/de OR 'diagnostic delay'/mj/de OR 'delayed diagnosis'/mj/de OR 'therapy delay'/mj/de OR 'health care utilization'/mj/de OR mortality/mj/exp OR morbidity/mj/exp OR 'health care quality'/mj/exp OR screening/mj OR 'mass screening'/exp/mj OR 'screening test'/mj OR 'palliative therapy'/exp/mj OR 'health care delivery'/mj OR 'health care access'/exp/mj OR 'surgical volume'/mj OR rehabilitation/exp/mj OR 'performance indicator'/de OR (impact* OR influence* OR affect* OR effect* OR exacerbate* OR (Disease NEAR/3 progression*) OR recur* OR avoid* OR postpon* OR post-pon* OR implication* OR ((diagnos* OR therap* OR treatment* OR

hospitali* OR services OR visit*) NEAR/3 (error* OR delay* OR fewer* OR drop OR decline* OR continuit* OR decrease* OR increase* OR reduc*)) OR ((health-care OR healthcare) NEAR/3 (utilizat* OR utilisat* OR use)) OR mortalit* OR morbidit* OR ((health-care OR healthcare) NEAR/3 (quality)) OR screening OR (service* NEAR/3 (disrupt* OR continu*)) OR palliat* OR unmet OR rehabilitation*):ti OR (((performance* OR outcome*) NEAR/3 (indicator*)) OR ((health-care OR healthcare) NEAR/3 (access* OR deliver* OR output*)) OR ((surgical OR surgeries OR procedures) NEAR/3 (volum* OR number*))):ab,ti) NOT (model/exp/mj OR (model*):ti) NOT ([conference abstract]/lim AND [2000-2019]/py) NOT ([animals]/lim NOT [humans]/lim) NOT ('health care personnel'/exp/mj OR pregnancy/exp/mj OR 'pregnant woman'/mj OR (((COVID-19 OR COVID19 OR coronavirus* OR corona-virus* OR SARS-CoV-2) NEAR/3 (outcome* OR case* OR patient* OR pneumoni* OR progression* OR prognos* OR mortalit* OR fatal* OR sever* OR vaccine*)) OR pregnan* OR ((healthcare OR health-care OR medical) NEAR/3 (personnel* OR staff OR worker* OR workforce* OR work-force*)) OR doctor* OR nurse* OR physician*):ti OR ((COVID-19 OR COVID19 OR coronavirus* OR corona-virus* OR SARS-CoV-2) NEAR/3 (mortalit* OR outcome* OR fatal*)):ab) NOT ((child/exp NOT adult/exp) OR (pediatr* OR paediatr* OR child* OR infan* OR adolescen*):ti) NOT ('practice guideline'/de OR (guideline*):ti)

Medline ALL Ovid 4429

(*Pandemics/ OR *Natural Disasters/ OR *Cyclonic Storms/ OR *Earthquakes/ OR *COVID-19/ OR *SARS-CoV-2/ OR (pandemic* OR disaster* OR hurricane* OR flood OR flooding OR earthquake* OR coronavirus-disease-2019 OR covid19 OR covid-19 OR sars-cov-2 OR 2019-novel-coronavirus OR 2019-ncov OR lockdown OR lock-down OR typhoon* OR (natural ADJ3 hazard*)).ti.) AND (Noncommunicable Diseases/ OR exp Neoplasms/ OR exp Heart Diseases/ OR Cardiology/ OR exp Nervous System Diseases/ OR Early Detection of Cancer/ OR exp Diabetes Mellitus/ OR exp Rheumatic Diseases/ OR exp Mental Health Services/ OR exp Cardiovascular Diseases/ OR exp Cerebrovascular Disorders/ OR exp Kidney Failure, Chronic/ OR Lung Diseases, Obstructive/ OR exp Stroke/ OR exp Hypertension/ OR Chronic Disease/ OR ((exp Emergency Medical Services/ OR exp Emergency Treatment/ OR General Practitioners/ OR General Practice/ OR Family Practice/) AND (Patient Acceptance of Health Care/ OR "Referral and Consultation"/)) OR (((non-communicab* OR noncommunicab*) ADJ3 disease) OR cancer OR ((heart OR cardiovascul* OR cerebrovascul*) ADJ3 (disease* OR patient* OR emergenc*)) OR cardiolog* OR neurolog* OR oncolog* OR ((((emergency OR acute) ADJ3 (ward* OR care OR department*)) OR (general ADJ (practi*)) OR ((primary OR family) ADJ2 (healthcare* OR care OR doctor*))) AND (utilization* OR utilisation* OR delay* OR time OR visit* OR consultation* OR barrier* OR access* OR challenge* OR vulnerab* OR attendan* OR hesita*)) OR diabet* OR rheumat* OR (mental ADJ3 (health-care* OR health-service* OR healthcare*)) OR (chronic ADJ3 (kidney OR renal OR respirator* OR lung* OR pulmonar*) ADJ3 (failure OR disease*)) OR asthma* OR bronchitis OR ((lung* OR pulmonar*) ADJ3 (emphysem*)) OR ((cerebrovascul* OR cerebro-vascul*) ADJ3 accident*) OR stroke OR hypertens* OR chronic-disease* OR (surg* ADJ3 emergenc*)).ab,ti.) AND (*Disease Progression/ OR exp * Recurrence/ OR *Undiagnosed Diseases/ OR exp *Diagnostic Errors/ OR *Delayed Diagnosis/ OR * Time-to-Treatment/ OR *Patient Acceptance of Health Care/ OR exp *Mortality/ OR exp *Morbidity/ OR exp

*Quality of Health Care/ OR *Early Detection of Cancer/ OR *Mass Screening/ OR *Palliative Care/ OR exp *Delivery of Health Care/ OR *Health Services Accessibility/ OR *Rehabilitation/ OR (impact* OR influence* OR affect* OR effect* OR exacerbate* OR (Disease ADJ3 progression*) OR recur* OR avoid* OR postpon* OR post-pon* OR implication* OR ((diagnos* OR therap* OR treatment* OR hospitali* OR services OR visit*) ADJ3 (error* OR delay* OR fewer* OR drop OR decline* OR continuit* OR decrease* OR increase* OR reduc*)) OR ((health-care OR healthcare) ADJ3 (utilizat* OR utilisat* OR "use")) OR mortalit* OR morbidit* OR ((health-care OR healthcare) ADJ3 (quality)) OR screening OR (service* ADJ3 (disrupt* OR continu*)) OR palliat* OR unmet OR rehabilitation*).ti. OR (((performance* OR outcome*) ADJ3 (indicator*)) OR ((health-care OR healthcare) ADJ3 (access* OR deliver* OR output*)) OR ((surgical OR surgeries OR procedures) ADJ3 (volum* OR number*))).ab,ti.) NOT (exp * Models, Theoretical/ OR model*:ti) NOT (exp animals/ NOT humans/) NOT (exp * Health Personnel/ OR exp * Attitude of Health Personnel / OR exp * pregnancy/ OR * pregnant woman/ OR (((COVID-19 OR COVID19 OR coronavirus* OR corona-virus* OR SARS-CoV-2) ADJ3 (outcome* OR case* OR patient* OR pneumoni* OR progression* OR prognos* OR mortalit* OR fatal* OR sever* OR vaccine*)) OR pregnan* OR ((healthcare OR health-care OR medical) ADJ3 (personnel* OR staff OR worker* OR workforce* OR work-force*)) OR doctor* OR nurse* OR physician*).ti. OR ((COVID-19 OR COVID19 OR coronavirus* OR corona-virus* OR SARS-CoV-2) ADJ3 (mortalit* OR outcome* OR fatal*)).ab.) NOT (((exp child/ OR exp infant/) NOT exp adult/) OR (pediatr* OR paediatr* OR child* OR infan* OR adolescen*).ti.) NOT (Practice Guideline/ OR Practice Guidelines as Topic/ OR (guideline*).ti.)

Supplementary material S3 – Data Extraction Form

Form 1: General study characteristics

Article ID	Number identifying each paper
Article source	Systematic search
	Reference mining
Clinical area	
Disease/care delivered	
Country	
Study design	Randomized controlled trial
	Prospective cohort
	Retrospective cohort
	Case-control
	Cross-sectional
	Case report
	Systematic review
	Meta-analysis
	Scoping review
	Survey
Peer-reviewed	Yes / No
Excluded: (with reason for exclusion)	Yes (reason) / No
Time period studied	
Comparison period	2020 vs. 2019
	2020: pre- versus post-COVID
	2020 vs. previous years
Article title	
Year of publication	
Journal	
Authors	
Paper URL	

Language	
Abstract	

Form 2: Indicator related information

Paper ID	Number identifying each paper
Clinical area	
Disease/care delivered	
Country	
Data source	Population-level data
	Adverse incident data
	Clinical data
	Costing data
	Claims data
	Survey data
	Registry data
	Prescribing data
	Claims data
Database	
Sample size	Number of centers
	Number of patients/procedures/responses/events
Indicator title	
Self-reported	Yes
	No
Numerator (inclusion criteria)	
Numerator (exclusion criteria)	
Denominator	
Indicator results from statistical model	Yes
	No
Periods being compared	2020 vs. 2019

	2020: pre- versus post-COVID
	2020 vs. previous years
Trend reported by indicator	Increase
	Stable
	Decrease
Numerical value before pandemic	
Numerical value during pandemic	
Units relating to numerical value	(eg: ng/L, days, admissions)
Magnitude (%)	*Computed as percent change:*<br>((value during pandemic - value before pandemic) /<br>(value before pandemic)) * 100
Main results (if reported)	Relative risk ratio
	Odds ratio
	Incidence rate ratio
Other results reported (free field)	
Delivery of care pathway	Access / admission
	Diagnostic
	Treatment
	Follow-up / Outpatient care
	Other
	Outcomes
Dimensions of performance	Access
	Quality – effectiveness
	Quality – safety
	Quality – patient-centredness
Donabedian's framework:	Structure
	Process
	Outcome

Supplementary material S4 – Collected and grouped indicators

Admission

Admission numbers	
**Indicator name**	**Number of papers**
Cardiovascular hospitalizations	3
Cardiology emergency admissions	2
Elective cardiology admissions	1
Admissions for acute cardiac events	1
Admitted to emergency department with cardiovascular symptoms	1
Admissions to cardiology ward / coronary care unit	1
Acute coronary syndrome (ACS) hospital admissions	13
Acute myorcardial infarction (STEMI/NSTEMI) hospital admissions	8
NSTEMI admissions	17
STEMI admissions	24
Unstable angina admissions	8
Heart failure hospital admissions	13
Admissions for cardiac arrhythmia	6
Admissions for out of hospital cardiac arrest	1
Valvular heart disease admissions	1
Myocarditis, pericarditis admissions	1
Number of hospitalizations for device malfunctioning	1
Threshold for hospitalization of heart failure patients	1

Delayed presentation	
**Indicator name**	**Number of papers**
ACS - Late presenting	1
STEMI - late presenting	11
NSTEMI - late presenting	2
AMI - self-presenting to hospital	4

Patient's clinical condition	
**Indicator name**	**Number of papers**
ACS - Left ventricular ejection fraction	2
ACS - Admission troponin	2
ACS - Admission CK	1
ACS - GRACE-score	1
ACS - Number of diseased vessels	3
ACS - Charlson comorbidity index	1
ACS - Cardiogenic shock	2
ACS - Life threatening arrhytmia	2
ACS - Intubation	1
ACS - Inotropic support	1
ACS - Resuscitation	1
ACS - Hemodynamic support	1
ACS - Invasive ventilation	1
AMI - Cardiogenic shock	1
STEMI patients admitted to ICU/CCU	2
STEMI - Ejection fraction at presentation	9
STEMI - Killip class at presentation	7
STEMI - Cardiogenic shock	8
STEMI - Cardiac arrest	8
STEMI - Asystole	1
STEMI - Ventricular fibrillation	2
STEMI - Mechanical ventilation	2
STEMI - Troponin	5
STEMI - LDH	2
STEMI - CK	2
STEMI - NTproBNP	1
STEMI - GRACE-score	1
STEMI - baseline TIMI-flow	7
STEMI - thrombosis degree	1
STEMI - Collateral circulation	1
STEMI - Number of vessels	8
STEMI - Presence of new Q-waves on the earliest ECG	1
STEMI - Atrial fibrillation	2
NSTEMI patients admitted to CCU	1
NSTEMI - GRACE-score	1
NSTEMI - Troponin	1
Heart failure - NYHA class	2
Heart failure - Charlson comorbidity index	3
Heart failure - LVEF	2
Heart failure - NT-proBNP	2
Heart failure - admission to ICU	3
Heart failure - SBP <100mmHg on admission	1
Heart failure - eGFR < 30	1
Acute heart failure patients admitted to ICU	1
Cardiac surgery - LVEF	1
Cardiac surgery - Preoperative EuroSCORE II	1
Cardiac surgery - patients admitted with a critical preoperative status	1
Patients admitted in cardiac intensive care unit - LVEF	1

Diagnostics

Diagnostics	
**Indicator name**	**Number of papers**
Number of angiographies	6
Number of SPECT-MPI	1
Number of cardiac imaging procedures	3
Number of non-invasive ischemia tests	2
Number of elective diagnostic catheterisation	1
Number of acute cardiac tests	1
Number of ECG's	1
Number of NT-proBNP blood tests in primary and secondary care	1
Number of dobutamine stress echocardiagram	1

Treatment

Procedure numbers	
**Indicator name**	**Number of papers**
Number of cardiac procedures performed	4
Number of PCI procedures	22
Number of transcatheter aortic valve replacements	4
Number of structural heart disease procedures	3
Number of mitraclip	1
Number of CABG procedures	5
Number of pacemaker/defibrillator implantations	4
Number of electrophysiology procedures	2
Number of cardioversions	2
Number of ablations	1
Number of cardiovascular surgeries	4
Heart failure - % of pts who underwent cardiac resynchronization therapy device implantation	1
Number of invasive coronary procedures because of ACS	1
Transplant cardiac activity	1

Length of stay	
**Indicator name**	**Number of papers**
Patients admitted to cardiology departments - length of stay	1
Patients admitted to cardiac intensive care unit/ICU - length of stay	5
Acute cardiovascular conditions - length of stay	1
Heart failure patients - average length of stay	3
Acute coronary syndrome (ACS) - length of stay	4
Acute myocardial infarction - length of stay	5
STEMI - length of stay	11
NSTEMI - length of stay	3
Postprocedure same-day discharge	1

ACS pathway times	
**Indicator name**	**Number of papers**
Symptom-to-contact	17
Symptom-to-door	9
Symptom-to-diagnosis	2
Symptom-to-balloon (ischaemia time)	14
Contact-to-door	3
Contact-to-balloon	10
Door-to-ECG	1
Door-to-balloon	24
ECG-to-balloon	2
Diagnosis-to-balloon	2
First medical contact to cath lab arrival	1
Cath-to-puncture	1
Cath to balloon	2
Puncture-to-balloon	1
Procedure time	3

Outpatient care

Outpatient activity & telehealth	
**Indicator name**	**Number of papers**
Outpatient activity	4
Referral to cardiac rehabilitation	3
Number of referrals to cardiology outpatient clinics	1
Number of in-person outpatient visits	2
Cancelled outpatient cardiology visits	2
Patients reporting that HF appointments were negatively impacted during the lockdown period	1
Outpatient care converted to tele-health	2
Proportion of telehealth visits	4

Outcomes

Patient outcomes and complications

Indicator name	Number of papers
LVEF <40% at discharge / <35% at discharge	3
Post revascularization left ventricular ejection fraction	2
Post infarction left ventricular ejection fraction	2
Left ventricular ejection fraction at discharge	4
Occurence of stent thrombosis	5
Cardiogenic shock / mechanical complication in STEMI	1
In-hospital re-PCI	2
Major adverse cardiovascular event (MACE)	5
Rates of emergency department / hospitalization 30 days post in-person visits	1
Cardiogenic shock / CPR after cardiovasular emergencies	1
ACS/STEMI patients developing mechanical complication	4
Troponin peak value	6
TIMI flow after PCI	11
STEMI patients - Pericardial effusion	1
STEMI patients - Bleeding requiring blood transfusion	2
STEMI patients - AKI / Renal failure / Dialysis	3
STEMI - Left ventricular aneurysm	1
Reinfarction	3
STEMI - Malignant arrhytmia	1
STEMI - Cardiogenic shock	1
STEMI - Target vessel urgent revascularization	1
STEMI - Stroke	2
STEMI patients - LV thrombus	2
STEMI - Procedural success	1
STEMI - Coronary perforation	1
STEMI - Retroperitoneal bleed	1
STEMI - Thrombo embolism	1
STEMI - Mechanical ventilation	1
STEMI - Inotropic support	1
STEMI - Mechanical circulatory support	1
STEMI - Sepsis	1
STEMI - Post PCI ECG, ST resolution >70%	1
STEMI - Post PCI CK	1
Number of deaths due to infections after PCI	1
AMI - NTproBNP	1
STEMI - TIMI frame count <23	1
30-day hospital readmission rate for heart failure	1
Rates of ED/hospitalization 30 days post In-person visits	1

Mortality rates

Indicator name	Number of papers
Intra-hospital cardiovascular mortality	2
In-hospital mortality of patients admitted to cardiology departments	1
Cardiac mortality	2
In-hospital mortality rate of acute coronary syndrome (STEMI, NSTEMI, unstable angina)	5
In-hospital mortality rate of acute myocardial infarction (STEMI, NSTEMI)	4
In-hospital mortality rate of NSTEMI	2
In-hospital mortality rate of STEMI	16
7-day mortality AMI patients	1
30-day mortality AMI patients	1
30-day death rate after PCI	1
In-hospital mortality in patients admitted for cardiac catherization/PCI	3
Postdischarge mortality up to 30 days after PCI	1
All-cause mortality STEMI patients	1
Rate of death at the emergency department for cardiovascular diseases	1
Death caused by ischemic heart disease	1
In-hospital death in patients admitted to CCU	1
In-hospital mortality among cardiac surgery recipients	1
Short-term (30 day) case mortality of patients diagnosed with acute HF	1
Death caused by heart failure	1
In-hospital mortality rate of heart failure patients	4

Supplementary material S5 – Characteristics of the studies included in the review, and from which indicators were extracted and collated

**Reference Number** (document with references extra)	**COUNTRY**	**Study design**	**Time period studied - Start**	**Time period studied - End**	**Comparison 2020 vs. 2019**	**Comparison 2020: pre / post Covid**	**Comparison 2020 vs. previous years**
1	Canada	Prospective + retrospective cohort	01-03-2020	19-04-2020	2019: same period		
2	Multiple countries (a)	Retrospective cohort	12-03-2020	15-04-2020		01-12-2019 to 11-03-2020	12-2018 to 04-2019
3	United Kingdom	Retrospective cohort	23-03-2020	10-05-2020		2018-2019 VS before 1st case: 28-10-2019 to 02-02-2020; 2018-2019 VS between 1stcase and lockdown: 03-02-2020 to 22-03-2020	2018-2019: corresponding dates
4	US	Retrospective cohort	01-01-2020	31-03-2020	2019: same period		
5	Switzerland	Retrospective cohort	01-01-2020	28-02-2020	2019: same period	Mar-Apr 2020	
6	Germany	Retrospective cohort	13-03-2020	30-04-2020	2019: same period	01-01-2020 to 12-03-2020	
7	United Kingdom	Retrospective cohort	02-03-2020	19-04-2020			2017-2019: same period
8	Israel	Retrospective cohort	01-02-2020	30-06-2020			01-07-2018 to 31-01-2020
9	Austria	Retrospective cohort	16-03-2020	26-04-2020		03-02-2020 to 15-03-2020	2016-2019: same period
10	Denmark	Prospective cohort	13-03-2020	16-10-2020	2019: same period		

11	Belgium	Retrospective cohort	13-03-2020	03-04-2020			2017-2019: same period
12	Italy	Retrospective cohort	20-02-2020	20-04-2020	2019: same period		
13	Italy	Retrospective cohort	20-02-2020	31-03-2020	2019: same period	01-01-2020 to 19-02-202	
14	United Kingdom	Retrospective cohort	03-03-2020	27-04-2020		07-01-2020 to 02-03-2020	
15	Turkey	Prospective cohort	17-04-2020	02-05-2020			2018: 1 to 15-11-2018
16	Italy	Retrospective cohort	01-03-2020	30-04-2020	2019: same period		2015-2019: same period
17	United Kingdom	Retrospective cohort	21-04-2020	20-05-2020		21-03-2020 to 20-04-2020 (also lockdown); 21-02 to 20-03-2020 (transition period); 21-01-2020 to 20-02-2020 (baseline)	
18	Italy	Retrospective cohort	10-03-2020	10-04-2020	2019: same period		
19	Italy	Retrospective cohort	24-02-2020	29-03-2020	2019: same period	8 weeks before	
20	Poland	Retrospective cohort	09-03-2020	16-04-2020	2019: same period		
21	Germany	Retrospective cohort	01-03-2020	21-04-2020	2019: same period		2017-2019: same period
22	US	Retrospective cohort	23-02-2020	28-03-2020		29-03-2020 to 16-05-2020	30-12-2018 to 22-02-2020
23	Italy	Prospective cohort	21-02-2020	01-04-2020	2019: same period		2018: same period
24	Switzerland	Retrospective cohort	17-02-2020	14-04-2020	2019: same period		
25	Greece	Prospective cohort	01-02-2020	30-06-2020		01-01-2019 to 31-01-2020	
26	Turkey	Retrospective cohort	10-03-2020	30-04-2020			2015-2019: same period
27	Germany	Retrospective cohort	01-03-2020	01-04-2020	2019: same period	01-01-2020 to 12-03-2020	

28	Germany	Retrospective cohort	13-03-2020	10-09-2020	2019: same period		
29	United Kingdom	Retrospective cohort	23-03-2020	24-05-2020	2019: weekly/monthly average	01-01-2019 to 22-03-2020	
30	US	Survey	30-04-2020	13-05-2020		"Compared to pre-Covid"	
31	Spain	Retrospective cohort	12-03-2020	20-04-2020		01-02-2020 to 11-03-2020	
32	Germany	Retrospective cohort	23-03-2020	26-04-2020	2019: same period	Jan-Feb 2020 VS Jan-Feb 2019 (adjacent non-pandemic period)	
33	Spain	Retrospective cohort	04-03-2020	19-04-2020		17-01-2020 to 03-03-2020	
34	Greece	Retrospective cohort	02-03-2020	12-04-2020	2019: same period		
35	Italy	Retrospective cohort	01-03-2020	31-03-2020	2019: same period		
36	Germany	Retrospective cohort	01-03-2020	30-04-2020	2019: same period	01-01-2020 to 29-02-2020	
37	Germany	Retrospective cohort	21-03-2020	20-04-2020			2017-2019: same period
38	Austria	Retrospective cohort	24-02-2020	05-04-2020		Comparison betweet the six weeks (1: calendar week 9-10, 2: week 11-12, 3: week 13-14)	
39	Multiple countries (b)	Survey	01-04-2020	15-04-2020			
40	Austria	Retrospective cohort	01-01-2020	31-05-2020	2019: same period		2017-2018: same period
41	Italy	Retrospective cohort	01-03-2020	31-03-2020	2019: same period		
42	Multiple countries (c)	Systematic review/ Pooled analysis			2019: same period	X	

43	Multiple countries (d)	Retrospective cohort	01-03-2020	30-04-2020	2019: same period, (1 month: each investigator decided the relevant time period in his country)		
44	US	Retrospective cohort	04-03-2020	14-04-2020	2019: same period	01-01-2020 to 03-03-2020	
45	Italy	Retrospective cohort	21-02-2020	10-04-2020		3-1-2020 to 20-02-2020	
46	Greece	Retrospective cohort	15-03-2020	14-04-2020	2019: same period		
47	Italy	Retrospective cohort + Literature review	09-03-2020	09-04-2020	2019: same period	Previous months of 2020	
48	US	Retrospective cohort	01-01-2020	26-04-2020	2019: same period		
49	United Kingdom	Retrospective cohort	23-03-2020	19-04-2020		01-01-2019 to 22-03-2020 (before Covid)	
50	Lithuania	Retrospective cohort	11-03-2020	20-04-2020	2019: same period		
51	US	Prospective cohort	23-03-2020	15-04-2020		01-01-2020 to 22-03-2020	
52	Japan	Retrospective cohort	07-04-2020	14-08-2020			01-01-2018 to 6-04-2020
53	Switzerland	Retrospective cohort	13-03-2020	30-04-2020	2019: same period	07-01-2020 to 24-02-2020	
54	Australia	Retrospective cohort	16-03-2020	15-04-2020			2014-2019: same period
55	United Kingdom	Retrospective cohort	19-02-2020	14-04-2020	2019: same period		2016-2018: same period
56	US	Survey	15-03-2020	15-04-2020	2019: same period		
57	Italy	Retrospective cohort	20-02-2020	14-04-2020	2019: same period		
58	United Kingdom	Retrospective cohort	16-03-2020	16-05-2020			2017-2019: same period

59	Multiple countries (e)	Retrospective cohort	01-03-2020	30-04-2020	2019: same period		
60	Italy	Survey	12-03-2020	19-03-2020	2019: same period		
61	Canada	Retrospective cohort	mid-March 2020	mid-May 2020	2019: same period	January to mid-March 2020	
62	US		15-03-2020	25-04-2020		01-01-2019 to 14-03-2020	
63	France	Retrospective cohort	17-02-2020	26-04-2020			2018-2019: same period
64	Israel	Prospective cohort	20-03-2020	30-04-2020	2019: same period		
65	United Kingdom	Retrospective cohort	01-02-2020	30-04-2020			2017-2019
66	United Kingdom	Retrospective cohort	01-03-2020	30-04-2020	2019: same period		
67	France	Prospective cohort	09-03-2020	10-05-2020	2019: same period		
68	Spain	Prospective cohort	01-01-2020	10-05-2020	2019: same period		
69	France	Prospective cohort	26-02-2020	10-03-2020			2008-2017
70	Spain	Retrospective cohort	16-03-2020	14-04-2020	2019: 01-04 to 30-04		
71	Germany	Prospective cohort	01-03-2020	31-03-2020			2017-2019: month of March
72	US	Retrospective cohort	01-03-2020	30-04-2020	01-2019 to 02-2020		
73	Italy	Prospective cohort	01-02-2020	31-05-2020	2019: same period		2017-2018: same period
74	Italy	Retrospective cohort	01-03-2020	20-04-2020	2019: same period		
75	Ireland	Prospective cohort	01-03-2020	30-04-2020	2019: same period	Jan-Feb 2020	
76	New Zealand	Prospective cohort	30-12-2019	03-05-2020	2019: same period		
77	Italy	Retrospective cohort	01-03-2020	31-05-2020	2019: same period		
78	Japan	Survey	15-04-2020	30-04-2020		3rd survey in 15-May	
79	United Kingdom	Retrospective cohort	23-03-2020	30-04-2020		01-01-2020 to 22-03-2020	2017-2019
80	United Kingdom	Retrospective cohort	01-01-2020	10-05-2020			2017-2019
81	Spain	Survey	01-01-2020	30-09-2020	2019: same period		

82	Turkey	Retrospective cohort	10-03-2020	not clear		before 13-01-2020 (not clear)	
83	Spain	Retrospective cohort	15-03-2020	25-04-2020			2015-2019: same period
84	Multiple countries (f)	Survey	01-03-2020	30-04-2020	2019: march		
85	Multiple countries (g)	Survey	15-06-2020	03-07-2020			
86	United Kingdom	Survey	15-06-2020	10-08-2020			
87	US	Retrospective cohort	15-03-2020	30-06-2020	2019: same period		
88	France	Survey	17-03-2020	27-03-2020			
89	US	Retrospective cohort	18-03-2020	02-06-2020	2019: same period	01-01-2020 to 07-03-2020	

a) 17 Countries: Argentina, Mexico, Italy, US, Egypt, Spain, Perú, Austria, France, Saudi Arabia, Serbia, Poland, Turkey, Greece, Romania, Portugal, Uzbekistan, UK, Libya

b) Various countries - 49% Western Europe, 21% Eastern Europe, 13% Africa/Middle East, 11% Australasia and 6% Americas

c) 22 studies Europe, 8 USA, 3 China, 2 UK, 1 Canada, 1 Australia.

d) European centers - Poland, The Netherlands, Sweden, Portugal, Switzerland, Italy, Hungary, UK, Germany, Finland, Denmark, France, Spain, US

e) 77 European centers (5 European geographical areas were identified-1: Italy; 2: Iberian Peninsula (Spain and Portugal); 3: Central Europe (France,Germany,The Netherlands,Belgium,Czech Republic); 4:Balkan Peninsula (Romania,Slovenia,Greece,North Macedonia);5:North-East Europe(UK,Poland,Finland,Denmark,Russia).

f) 108 countries (Centers: Africa 42, Eastern Europe: 62, Far East: 99, Latin America: 201, MiddleEast/S.Asia: 60, US,Canada: 172, SoutheastAsia/Pacific:81, Western Europe: 192)

g) 23 countries (Excellence Center network of the European Society of Hypertension) - 20 European, 2Latin American,1Middle Eastern: (Italy,Greece,Spain,United Kingdom,Hungary,Austria, Brazil, France, Germany,Serbia, Sweden, Argentina,Armenia,Belgium,Bulgaria, Czech Republic, Estonia, Finland, Lebanon,Poland, Romania, Slovenia and Ukraine)

Supplementary material S6 – Number of indicators and indicators' trends reported by each indicator, presented by country

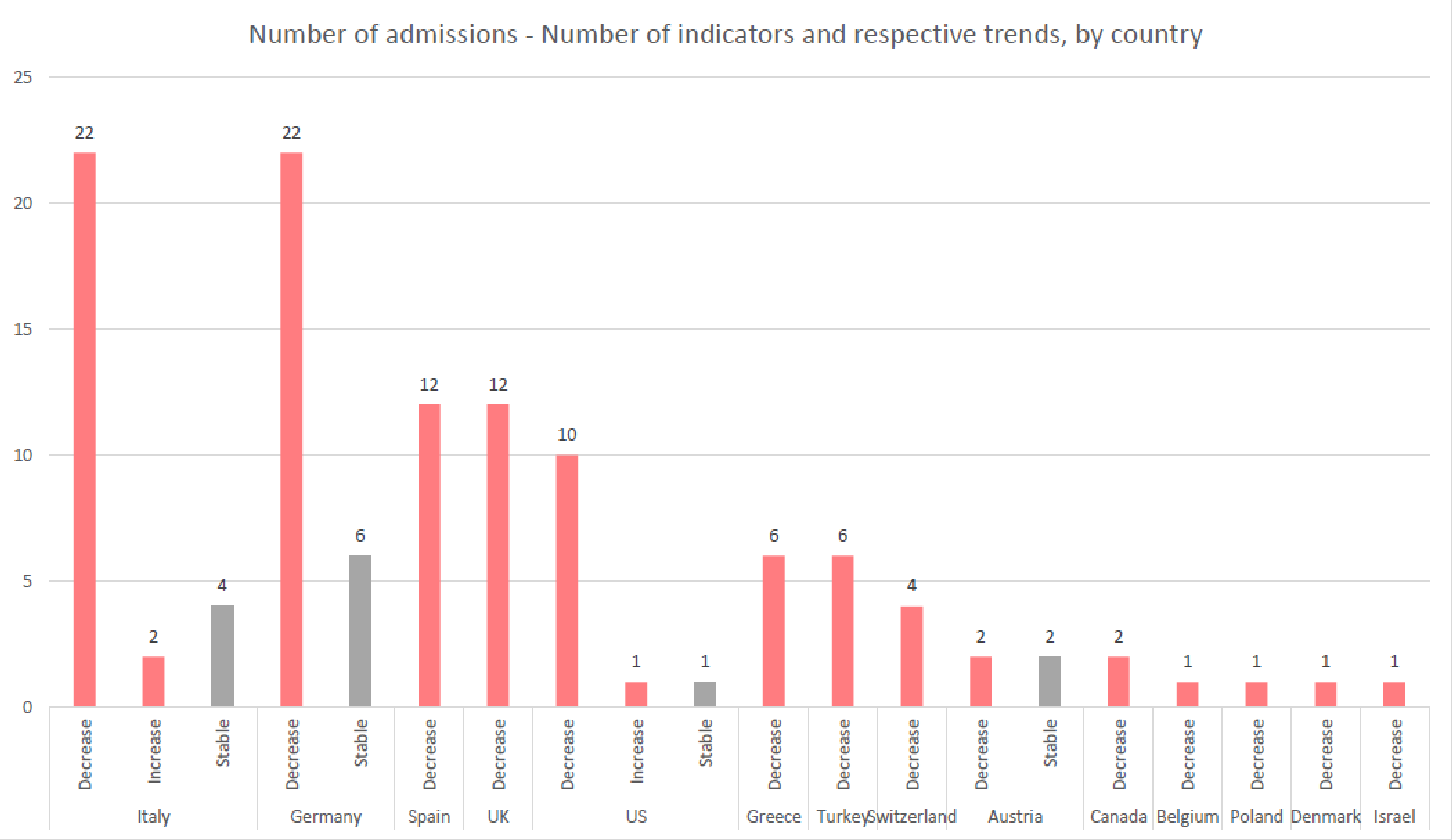

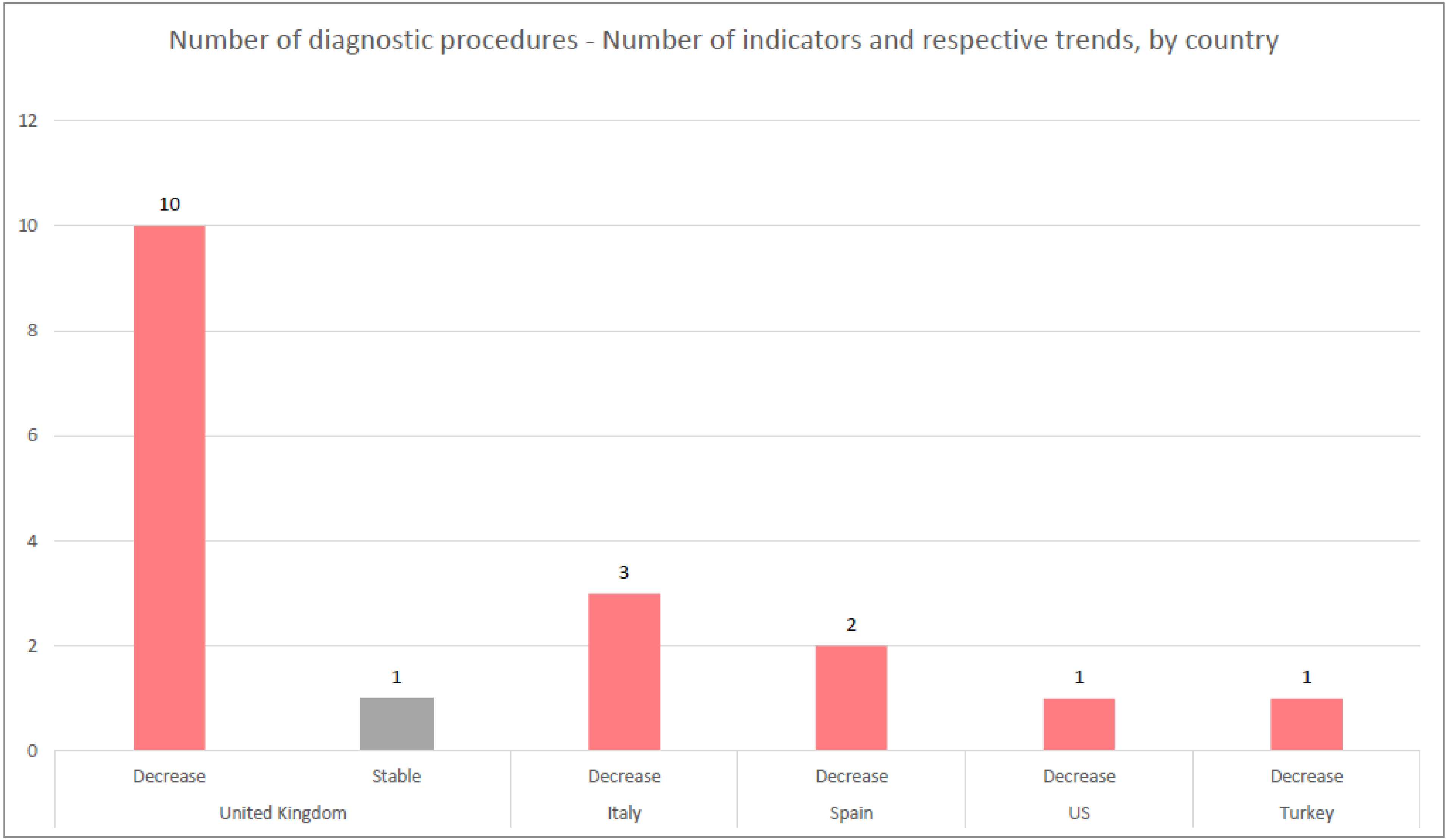
Number of diagnostic procedures - Number of indicators and respective trends, by country
12
10
8
6
4
2
0
10
1
3
2
1
1
Decrease
Stable
Decrease
Decrease
Decrease
Decrease
United Kingdom
Italy
Spain
US
Turkey

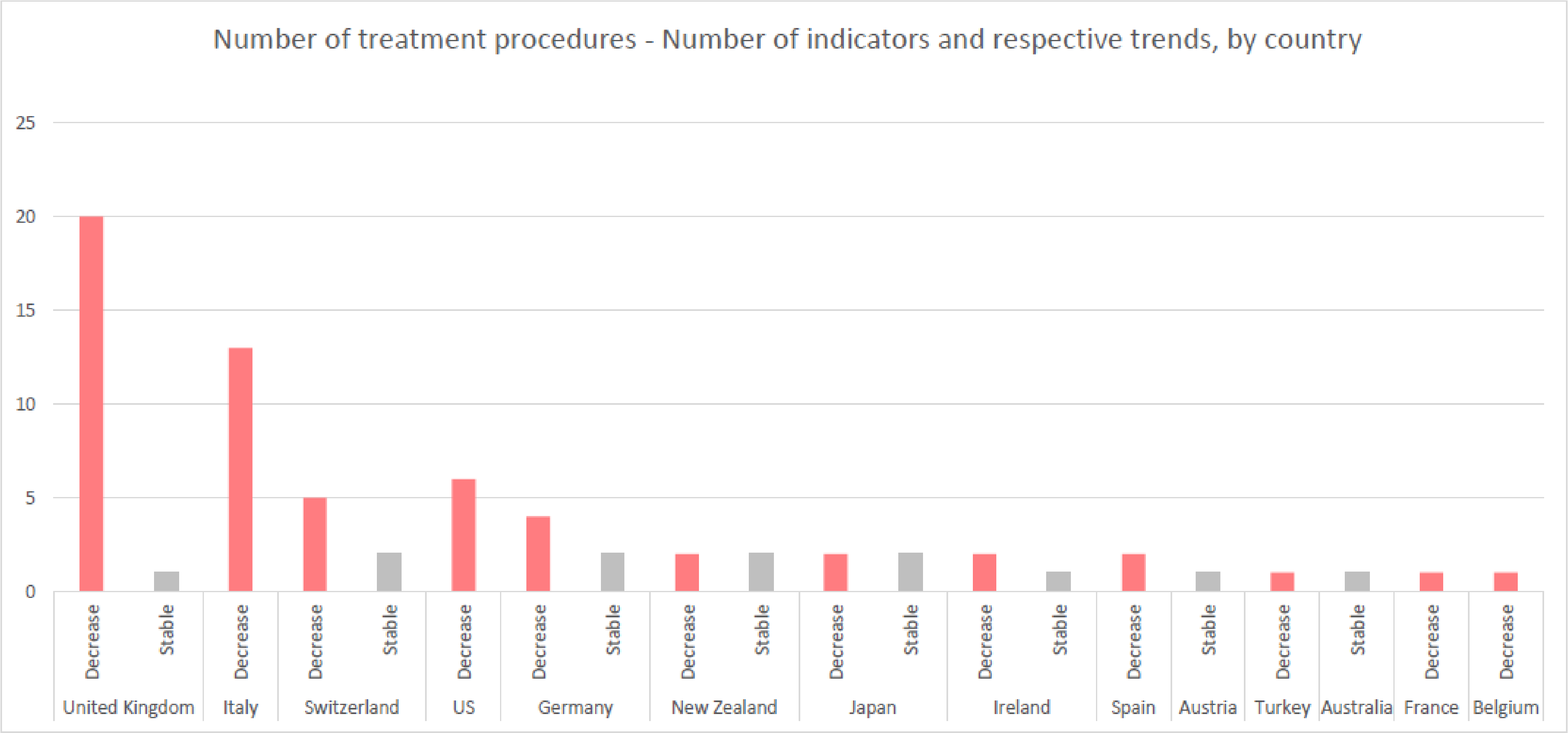
Number of treatment procedures - Number of indicators and respective trends, by country
25
20
15
10
5
0
Decrease
Stable
United Kingdom
Decrease
Italy
Decrease
Stable
Switzerland
Decrease
US
Decrease
Stable
Germany
Decrease
Stable
New Zealand
Decrease
Stable
Japan
Decrease
Stable
Ireland
Decrease
Spain
Stable
Austria
Decrease
Turkey
Stable
Australia
Decrease
France
Decrease
Belgium

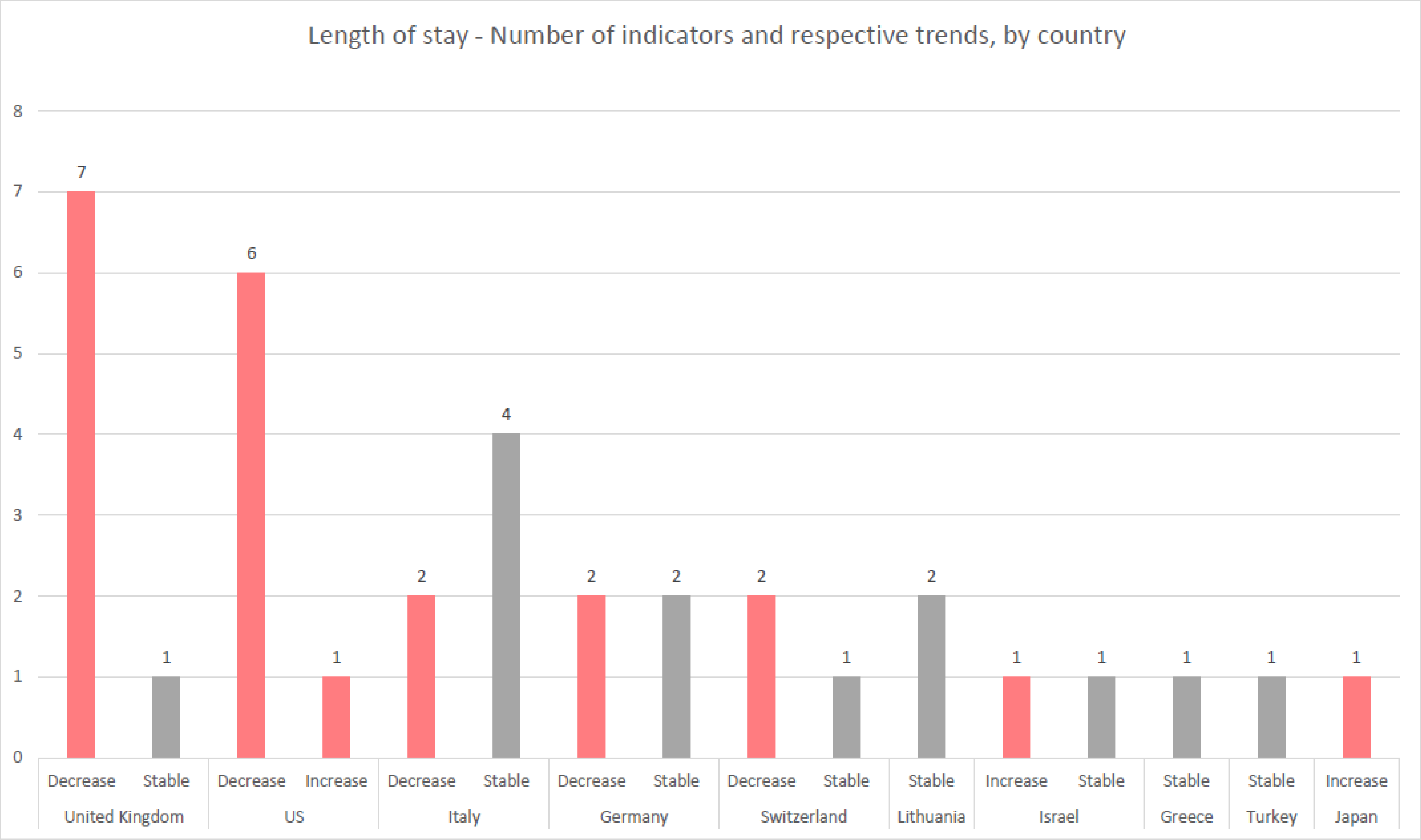
Length of stay - Number of indicators and respective trends, by country
8
7
6
5
4
3
2
1
0
7
1
6
1
2
4
2
2
2
1
2
1
1
1
1
1
Decrease
Stable
Decrease
Increase
Decrease
Stable
Decrease
Stable
Decrease
Stable
Stable
Increase
Stable
Stable
Stable
Increase
United Kingdom
US
Italy
Germany
Switzerland
Lithuania
Israel
Greece
Turkey
Japan

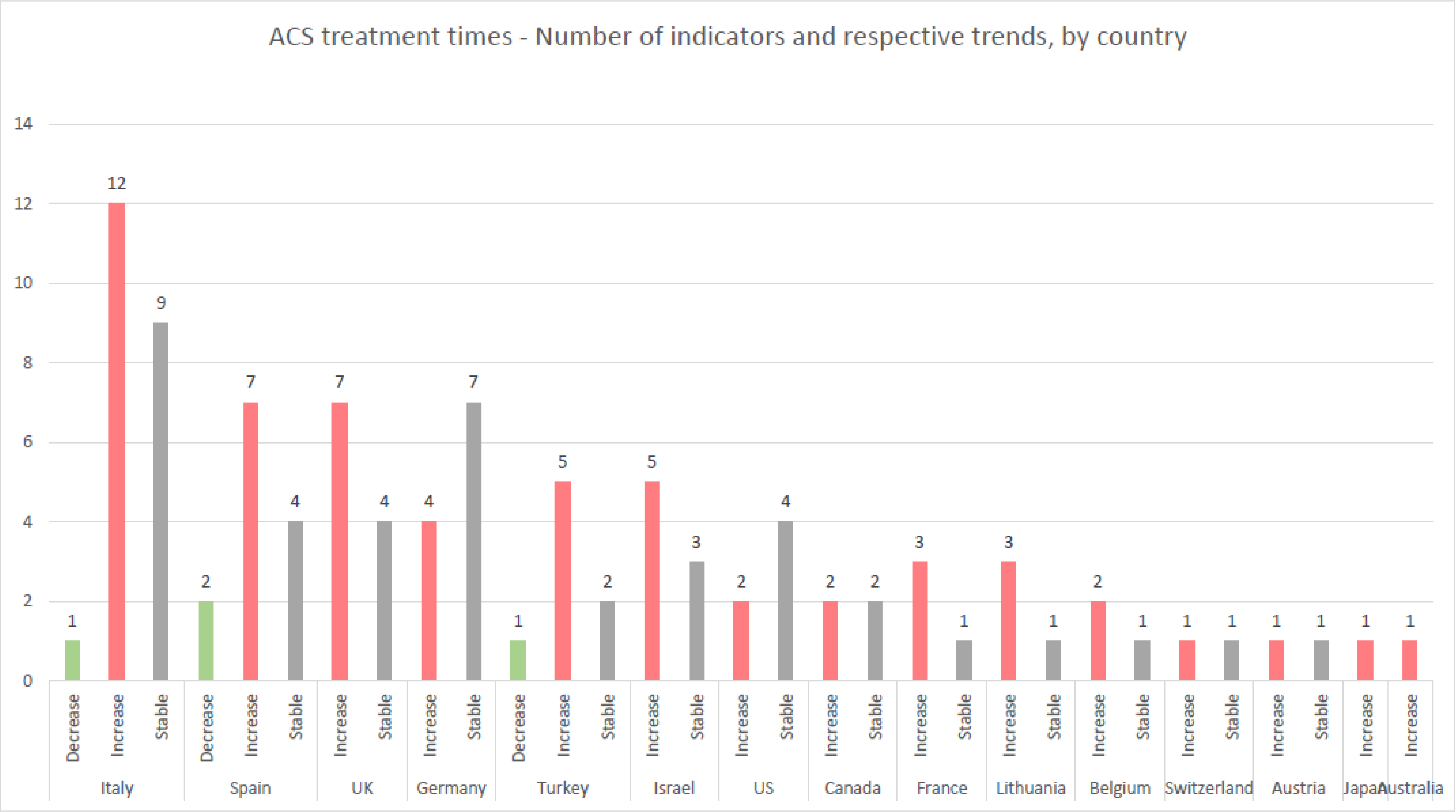
ACS treatment times - Number of indicators and respective trends, by country
14
12
10
8
6
4
2
0
1
12
9
2
7
4
7
4
4
7
1
5
2
5
3
2
4
2
2
3
1
3
1
2
1
1
1
1
1
1
1
Decrease
Increase
Stable
Italy
Spain
UK
Germany
Turkey
Israel
US
Canada
France
Lithuania
Belgium
Switzerland
Austria
Japa
Australia

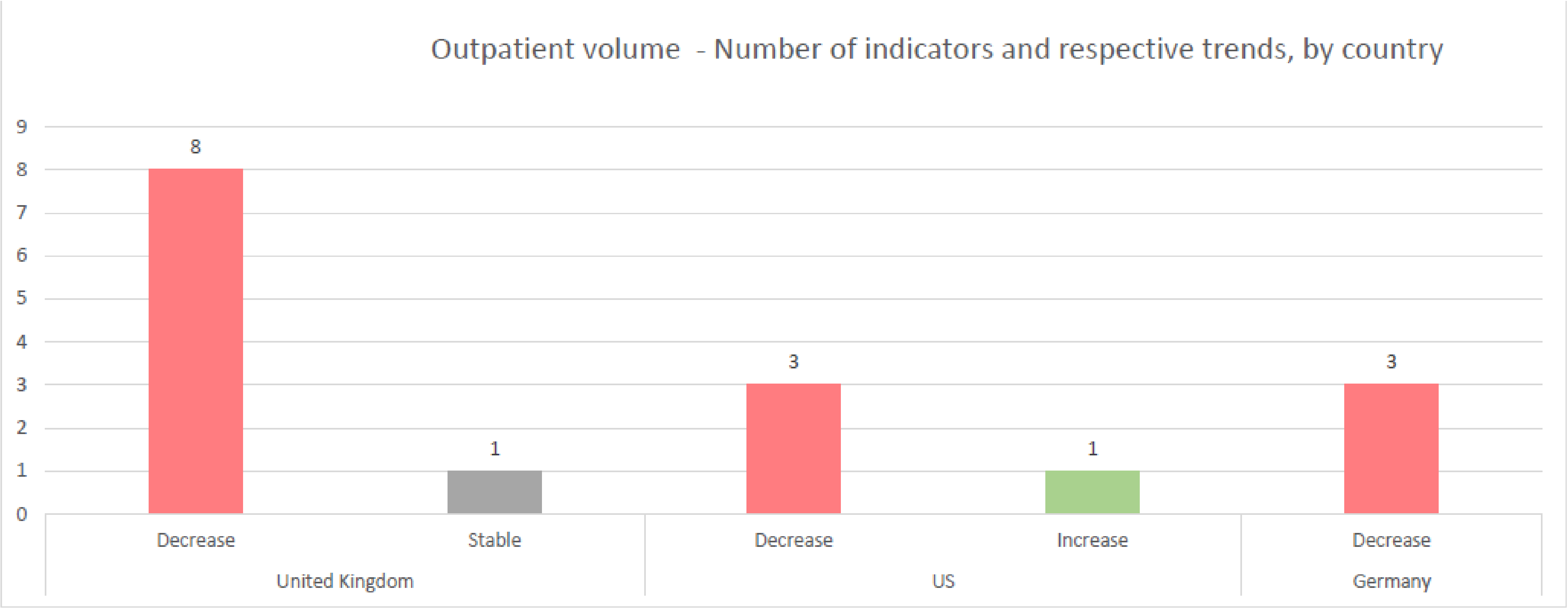
Outpatient volume - Number of indicators and respective trends, by country
9
8
7
6
5
4
3
2
1
0
8
1
3
1
3
Decrease
Stable
Decrease
Increase
Decrease
United Kingdom
US
Germany

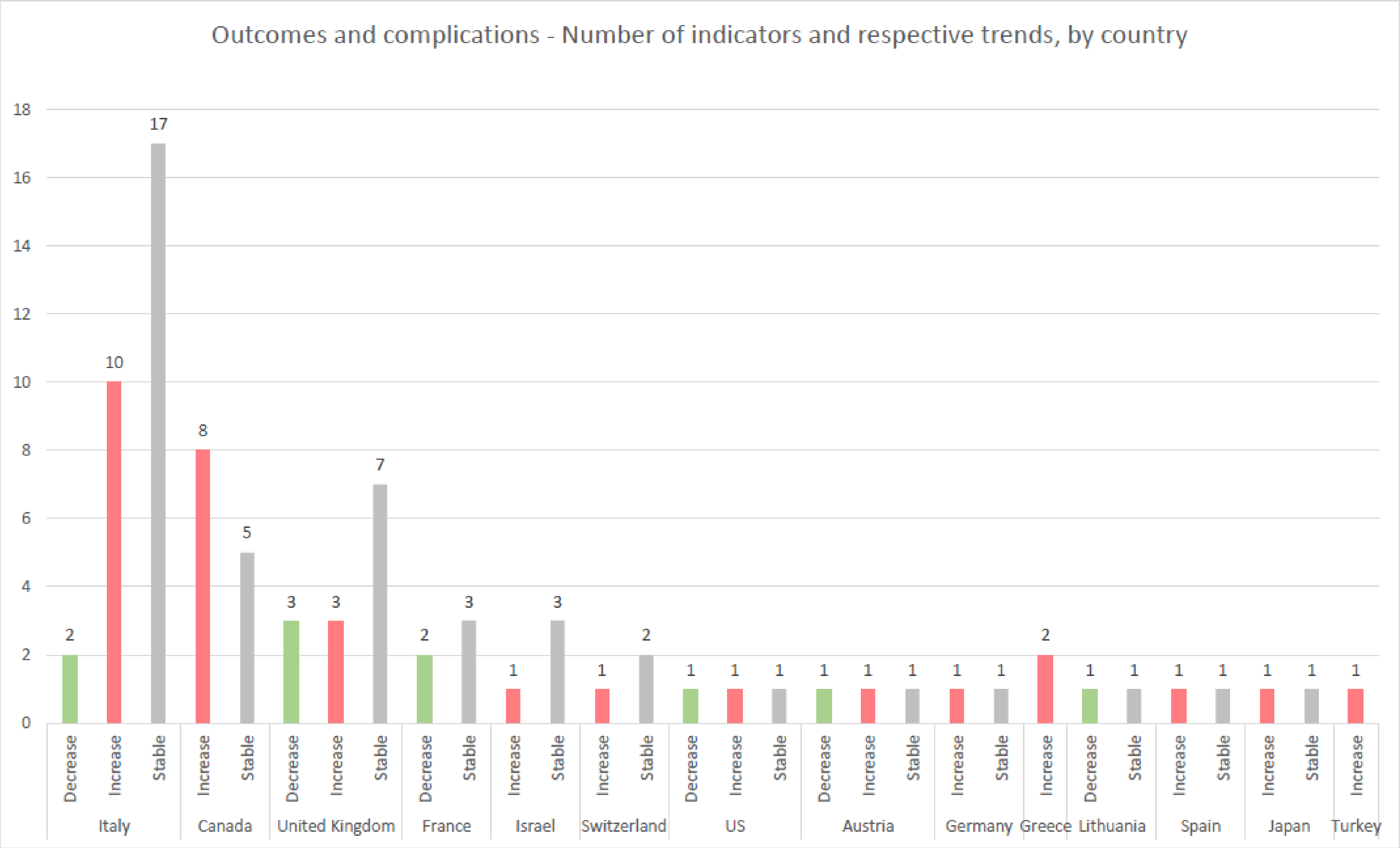
Outcomes and complications - Number of indicators and respective trends, by country
18
16
14
12
10
8
6
4
2
0
2
10
17
8
5
3
3
7
2
3
1
3
1
2
1
1
1
1
1
1
1
1
2
1
1
1
1
1
1
1
Decrease
Increase
Stable
Increase
Stable
Decrease
Increase
Stable
Decrease
Stable
Increase
Stable
Increase
Stable
Decrease
Increase
Stable
Decrease
Increase
Stable
Increase
Stable
Increase
Decrease
Stable
Increase
Stable
Increase
Stable
Increase
Italy
Canada
United Kingdom
France
Israel
Switzerland
US
Austria
Germany
Greece
Lithuania
Spain
Japan
Turkey

Supplementary material S7 – Additional references: studies included in the review, and from which indicators were extracted and collated

1. Alba A, Frankfurter C, Buchan T et al. REDUCED RATE OF HOSPITAL PRESENTATIONS FOR HEART FAILURE DURING THE COVID-19 PANDEMIC IN TORONTO, CANADA. *Can J Cardiol* 2020;36:S74. https://www.ncbi.nlm.nih.gov/pmc/articles/PMC7529371/ 10.1016/j.cjca.2020.07.147

2. Araiza-Garaygordobil D, Montalto C, Martinez-Amezcua P et al. Impact of the COVID-19 Pandemic on Hospitalizations for Acute Coronary Syndromes: a Multinational Study. *QJM* 2021;10.1093/qjmed/hcab013

3. Ball S, Banerjee A, Berry C et al. Monitoring indirect impact of COVID-19 pandemic on services for cardiovascular diseases in the UK. *Heart* 2020;106:1890-1897.10.1136/heartjnl-2020-317870

4. Bhatt AS, Moscone A, McElrath EE et al. Fewer Hospitalizations for Acute Cardiovascular Conditions During the COVID-19 Pandemic. *J Am Coll Cardiol* 2020;76:280-288.10.1016/j.jacc.2020.05.038

5. Boeddinghaus J, Nestelberger T, Kaiser C et al. Effect of COVID-19 on acute treatment of ST-segment elevation and Non-ST-segment elevation acute coronary syndrome in northwestern Switzerland. *Int J Cardiol Heart Vasc* 2021;32:100686.10.1016/j.ijcha.2020.100686

6. Bollmann A, Hohenstein S, König S, Meier-Hellmann A, Kuhlen R, Hindricks G. In-hospital mortality in heart failure in Germany during the Covid-19 pandemic. *ESC Heart Failure* 2020;7:4416-4419. https://onlinelibrary.wiley.com/doi/abs/10.1002/ehf2.13011 10.1002/ehf2.13011

7. Bromage DI, Cannatà A, Rind IA et al. The impact of COVID-19 on heart failure hospitalization and management: report from a Heart Failure Unit in London during the peak of the pandemic. *European Journal of Heart Failure* 2020;22:978-984. https://onlinelibrary.wiley.com/doi/abs/10.1002/ejhf.1925 10.1002/ejhf.1925

8. Bruoha S, Yosefy C, Gallego-Colon E et al. Impact in total ischemic time and ST-segment elevation myocardial infarction admissions during COVID-19. *Am J Emerg Med* 2021;45:7-10.10.1016/j.ajem.2021.02.020

9. Bugger H, Gollmer J, Pregartner G et al. Complications and mortality of cardiovascular emergency admissions during COVID-19 associated restrictive measures. *PLoS One* 2020;15:e0239801.10.1371/journal.pone.0239801 38

10. Butt JH, Fosbøl EL, Gerds TA et al. All-cause mortality and location of death in patients with established cardiovascular disease before, during, and after the COVID-19 lockdown: a Danish Nationwide Cohort Study. *European Heart Journal* 2021;42:1516-1523. https://doi.org/10.1093/eurheartj/ehab028 10.1093/eurheartj/ehab028

11. Claeys MJ, Argacha J, Collart P et al. Impact of COVID-19-related public containment measures on the ST elevation myocardial infarction epidemic in Belgium: a nationwide, serial, cross-sectional study. *Acta Cardiol* 2020;:1-7.10.1080/00015385.2020.1796035

12. Colivicchi F, Di Fusco SA, Magnanti M, Cipriani M, Imperoli G. The Impact of the Coronavirus Disease-2019 Pandemic and Italian Lockdown Measures on Clinical Presentation and Management of Acute Heart Failure. *J Card Fail* 2020;26:464-465. https://www.ncbi.nlm.nih.gov/pmc/articles/PMC7224656/ 10.1016/j.cardfail.2020.05.007

13. De Filippo O, D'Ascenzo F, Angelini F et al. Reduced Rate of Hospital Admissions for ACS during Covid-19 Outbreak in Northern Italy. *N Engl J Med* 2020;383:88-89.10.1056/NEJMc2009166

14. Doolub G, Wong C, Hewitson L et al. Impact of COVID-19 on inpatient referral of acute heart failure: a single-centre experience from the south-west of the UK. *ESC Heart Failure* 2021;8:1691-1695. https://onlinelibrary.wiley.com/doi/abs/10.1002/ehf2.13158 10.1002/ehf2.13158

15. Erol MK, Kayıkçıoğlu M, Kılıçkap M et al. Treatment delays and in-hospital outcomes in acute myocardial infarction during the COVID-19 pandemic: A nationwide study. *Anatol J Cardiol* 2020;24:334-342.10.14744/AnatolJCardiol.2020.98607

16. Fabris E, Bessi R, De Bellis A et al. COVID-19 impact on ST-elevation myocardial infarction incidence rate in a Italian STEMI network: a U-shaped curve phenomenon. *Journal of Cardiovascular Medicine* 2021;22:344–349. https://journals.lww.com/jcardiovascularmedicine/Fulltext/2021/05000/COVID_19_impact_on_ST_elevation_myocardial.3.aspx 10.2459/JCM.0000000000001153

17. Fersia O, Bryant S, Nicholson R et al. The impact of the COVID-19 pandemic on cardiology services. *Open Heart* 2020;710.1136/openhrt-2020-001359

18. Fileti L, Vecchio S, Moretti C et al. Impact of the COVID-19 pandemic on coronary invasive procedures at two Italian high-volume referral centers. *J Cardiovasc Med (Hagerstown)* 2020;21:869-873.10.2459/JCM.0000000000001101

19. Folino AF, Zorzi A, Cernetti C et al. Impact of COVID-19 epidemic on coronary care unit accesses for acute coronary syndrome in Veneto region, Italy. *Am Heart J* 2020;226:26-28. https://www.ncbi.nlm.nih.gov/pmc/articles/PMC7201234/ 10.1016/j.ahj.2020.04.021

20. Gąsior M, Gierlotka M, Tycińska A et al. Effects of the coronavirus disease 2019 pandemic on the number of hospitalizations for myocardial infarction: regional differences. Population analysis of 7 million people. *Kardiol Pol* 2020;78:1039-1042.10.33963/KP.15559 39

21. Gitt AK, Karcher AK, Zahn R, Zeymer U. Collateral damage of COVID-19-lockdown in Germany: decline of NSTE-ACS admissions. *Clin Res Cardiol* 2020;:1-3. https://www.ncbi.nlm.nih.gov/pmc/articles/PMC7351542/ 10.1007/s00392-020-01705-x

22. Gluckman TJ, Wilson MA, Chiu S et al. Case Rates, Treatment Approaches, and Outcomes in Acute Myocardial Infarction During the Coronavirus Disease 2019 Pandemic. *JAMA Cardiol* 2020;5:1419-1424.10.1001/jamacardio.2020.3629

23. Gramegna M, Baldetti L, Beneduce A et al. ST-Segment-Elevation Myocardial Infarction During COVID-19 Pandemic: Insights From a Regional Public Service Healthcare Hub. *Circ Cardiovasc Interv* 2020;13:e009413.10.1161/CIRCINTERVENTIONS.120.009413

24. Holy EW, Jakob P, Manka R et al. Impact of a nationwide COVID-19 lockdown on acute coronary syndrome referrals. *Cardiol J* 2020;27:633-635. https://www.ncbi.nlm.nih.gov/pmc/articles/PMC8078978/ 10.5603/CJ.a2020.0091

25. Kapelios CJ, Siafarikas C, Bonou M, Liatis S, Barbetseas J. The effect of the COVID-19 pandemic on acute coronary syndrome hospitalizations and out-of-hospital cardiac arrest in Greece. *Public Health* 2021;191:17-19.10.1016/j.puhe.2020.12.006

26. Khalil E, Ozcan S. Comparison of the number of cardiovascular admissions before and after covid-19: Experience from Turkey. *Acta Medica Mediterranea* 2020;:2433-2437. https://doi.org/10.19193/0393-6384_2020_4_378

27. König S, Hohenstein S, Meier-Hellmann A, Kuhlen R, Hindricks G, Bollmann A. In-hospital care in acute heart failure during the COVID-19 pandemic: insights from the German-wide Helios hospital network. *Eur J Heart Fail* 2020;22:2190-2201.10.1002/ejhf.2044

28. König S, Ueberham L, Pellissier V et al. Hospitalization deficit of in- and outpatient cases with cardiovascular diseases and utilization of cardiological interventions during the COVID-19 pandemic: Insights from the German-wide helios hospital network. *Clinical cardiology* 2021;44:392-400. https://onlinelibrary.wiley.com/doi/abs/10.1002/clc.23549 10.1002/clc.23549

29. Mafham MM, Spata E, Goldacre R et al. COVID-19 pandemic and admission rates for and management of acute coronary syndromes in England. *The Lancet* 2020;396:381-389. https://www.thelancet.com/journals/lancet/article/PIIS0140-6736(20)31356-8/abstract 10.1016/S0140-6736(20)31356-8

30. MCILVENNAN CK, ALLEN LA, DEVORE AD, GRANGER CB, KALTENBACH LA, GRANGER BB. Changes in Care Delivery for Patients With Heart Failure During the COVID-19 Pandemic: Results of a Multicenter Survey. *J Card Fail* 2020;26:635-636. https://www.ncbi.nlm.nih.gov/pmc/articles/PMC7272147/ 10.1016/j.cardfail.2020.05.019

31. Moreno R, Alonso JJ, Caballero R et al. Age and Gender influence on time of arrival for STEMI patients during Covid-19 pandemic. *Am J Emerg Med* 2021;42:244-245. https://www.ncbi.nlm.nih.gov/pmc/articles/PMC7274966/ 10.1016/j.ajem.2020.06.013

32. Nef HM, Elsässer A, Möllmann H et al. Impact of the COVID-19 pandemic on cardiovascular mortality and catherization activity during the lockdown in central Germany: an observational study. *Clin Res Cardiol* 2021;110:292-301.10.1007/s00392-020-01780-0 40

33. Negreira Caamaño M, Piqueras Flores J, Mateo Gómez C. Impact of COVID-19 pandemic in cardiology admissions. *Med Clin (Engl Ed)* 2020;155:179-180. https://www.ncbi.nlm.nih.gov/pmc/articles/PMC7381901/ 10.1016/j.medcle.2020.05.006

34. Papafaklis MI, Katsouras CS, Tsigkas G et al. "Missing" acute coronary syndrome hospitalizations during the COVID-19 era in Greece: Medical care avoidance combined with a true reduction in incidence? *Clin Cardiol* 2020;43:1142-1149.10.1002/clc.23424

35. Pinto RD, Ferri C, Mammarella L et al. Increased cardiovascular death rates in a COVID-19 low prevalence area. *The Journal of Clinical Hypertension* 2020;22:1932-1935. https://onlinelibrary.wiley.com/doi/abs/10.1111/jch.14013 10.1111/jch.14013

36. Primessnig U, Pieske BM, Sherif M. Increased mortality and worse cardiac outcome of acute myocardial infarction during the early COVID-19 pandemic. *ESC Heart Failure* 2021;8:333-343. https://onlinelibrary.wiley.com/doi/abs/10.1002/ehf2.13075 10.1002/ehf2.13075

37. Rattka M, Baumhardt M, Dreyhaupt J et al. 31 days of COVID-19-cardiac events during restriction of public life-a comparative study. *Clinical research in cardiology* 2020;109:1476-1482. https://www.ncbi.nlm.nih.gov/pubmed/32494921 10.1007/s00392-020-01681-2

38. Reinstadler SJ, Reindl M, Lechner I et al. Effect of the COVID-19 Pandemic on Treatment Delays in Patients with ST-Segment Elevation Myocardial Infarction. *J Clin Med* 2020;9 https://www.ncbi.nlm.nih.gov/pmc/articles/PMC7408681/ 10.3390/jcm9072183

39. Roffi M, Capodanno D, Windecker S, Baumbach A, Dudek D. Impact of the COVID-19 pandemic on interventional cardiology practice: results of the EAPCI survey. *EuroIntervention* 2020;16:247-250.10.4244/EIJ-D-20-00528

40. Schnaubelt S, Domanovits H, Niederdoeckl J et al. The Impact of the COVID-19 Pandemic on Incidences of Atrial Fibrillation and Electrical Cardioversion at a Tertiary Care Emergency Department: An Inter- and Intra-year Analysis. *Front Med (Lausanne)* 2020;7:595881.10.3389/fmed.2020.595881

41. Secco GG, Zocchi C, Parisi R et al. Decrease and Delay in Hospitalization for Acute Coronary Syndromes During the 2020 SARS-CoV-2 Pandemic. *Canadian Journal of Cardiology* 2020;36:1152-1155. https://www.sciencedirect.com/science/article/pii/S0828282X2030502X 10.1016/j.cjca.2020.05.023

42. Singh S, Fong HK, Desai R, Zwinderman AH. Impact of COVID-19 on acute coronary syndrome-related hospitalizations: A pooled analysis. *Int J Cardiol Heart Vasc* 2021;32 https://www.ncbi.nlm.nih.gov/pmc/articles/PMC7836359/ 10.1016/j.ijcha.2021.100718

43. Sokolski M, Gajewski P, Zymliński R et al. Impact of Coronavirus Disease 2019 (COVID-19) Outbreak on Acute Admissions at the Emergency and Cardiology Departments Across Europe. *Am J Med* 2021;134:482-489.10.1016/j.amjmed.2020.08.043

44. Solomon MD, McNulty EJ, Rana JS et al. The Covid-19 Pandemic and the Incidence of Acute Myocardial Infarction. *N Engl J Med* 2020;383:691-693.10.1056/NEJMc2015630 41

45. Tomasoni D, Adamo M, Italia L et al. Impact of COVID-2019 outbreak on prevalence, clinical presentation and outcomes of ST-elevation myocardial infarction. *J Cardiovasc Med (Hagerstown)* 2020;21:874-881.10.2459/JCM.0000000000001098
46. Vassilikos VP, Pagourelias ED, Katsos K et al. Impact of social containment measures on cardiovascular admissions and sudden cardiac death rates during Coronavirus Disease (COVID-19) outbreak in Greece. *Hellenic J Cardiol* 2020; https://www.ncbi.nlm.nih.gov/pmc/articles/PMC7491429/ 10.1016/j.hjc.2020.09.009
47. Vecchio S, Fileti L, Reggi A, Moschini C, Lorenzetti S, Rubboli A. Impact of the COVID-19 pandemic on admissions for acute coronary syndrome: review of the literature and single-center experience. *G Ital Cardiol (Rome)* 2020;21:502-508.10.1714/3386.33635
48. Vest AR, Upshaw JN, Dean K et al. The Decrease in Hospitalizations For Heart Failure During the Covid-19 Pandemic: A Community and Academic Hospital Comparison Study. *J Card Fail* 2020;26:S71. https://www.ncbi.nlm.nih.gov/pmc/articles/PMC7527197/ 10.1016/j.cardfail.2020.09.207
49. Wu J, Mamas M, Rashid M et al. Patient response, treatments, and mortality for acute myocardial infarction during the COVID-19 pandemic. *European Heart Journal - Quality of Care and Clinical Outcomes* 2021;7:238-246. https://doi.org/10.1093/ehjqcco/qcaa062 10.1093/ehjqcco/qcaa062
50. Aldujeli A, Hamadeh A, Briedis K et al. Delays in Presentation in Patients With Acute Myocardial Infarction During the COVID-19 Pandemic. *Cardiol Res* 2020;11:386-391.10.14740/cr1175
51. Hammad TA, Parikh M, Tashtish N et al. Impact of COVID-19 pandemic on ST-elevation myocardial infarction in a non-COVID-19 epicenter. *Catheter Cardiovasc Interv* 2021;97:208-214.10.1002/ccd.28997
52. Kitahara S, Fujino M, Honda S et al. COVID-19 pandemic is associated with mechanical complications in patients with ST-elevation myocardial infarction. *Open Heart* 2021;810.1136/openhrt-2020-001497
53. Perrin N, Iglesias JF, Rey F et al. Impact of the COVID-19 pandemic on acute coronary syndromes. *Swiss Med Wkly* 2020;150:w20448.10.4414/smw.2020.20448
54. Toner L, Koshy AN, Hamilton GW, Clark D, Farouque O, Yudi MB. Acute coronary syndromes undergoing percutaneous coronary intervention in the COVID-19 era: comparable case volumes but delayed symptom onset to hospital presentation. *Eur Heart J Qual Care Clin Outcomes* 2020;6:225-226.10.1093/ehjqcco/qcaa038
55. Wilson SJ, Connolly MJ, Elghamry Z et al. Effect of the COVID-19 Pandemic on ST-Segment-Elevation Myocardial Infarction Presentations and In-Hospital Outcomes. *Circ Cardiovasc Interv* 2020;13:e009438.10.1161/CIRCINTERVENTIONS.120.009438
56. Yong CM, Ang L, Welt FGP et al. Cardiac procedural deferral during the coronavirus (COVID-19) pandemic. *Catheter Cardiovasc Interv* 2020;96:1080-1086.10.1002/ccd.29262 42

57. Barbieri L, Tumminello G, Lucreziotti S et al. Mortality in STEMI Patients During the COVID Era: Has the Pandemic Changed Our Clinical Practice? *Cardiovasc Revasc Med* 2021;22:120-121. https://www.ncbi.nlm.nih.gov/pmc/articles/PMC7488593/ 10.1016/j.carrev.2020.09.014

58. Chen Y, Rathod KS, Hamshere S et al. COVID-19 and changes in activity and treatment of ST elevation MI from a UK cardiac centre. *Int J Cardiol Heart Vasc* 2021;33:100736.10.1016/j.ijcha.2021.100736

59. De Luca G, Cercek M, Jensen LO et al. Impact of COVID-19 pandemic and diabetes on mechanical reperfusion in patients with STEMI: insights from the ISACS STEMI COVID 19 Registry. *Cardiovasc Diabetol* 2020;19:215.10.1186/s12933-020-01196-0

60. De Rosa S, Spaccarotella C, Basso C et al. Reduction of hospitalizations for myocardial infarction in Italy in the COVID-19 era. *Eur Heart J* 2020;41:2083-2088.10.1093/eurheartj/ehaa409

61. Haddad K, Potter BJ, Matteau A, Gobeil F, Mansour S. Implications of COVID-19 on Time-Sensitive STEMI Care: A Report From a North American Epicenter. *Cardiovasc Revasc Med* 2021;30:33-37.10.1016/j.carrev.2020.09.024

62. Hannan EL, Wu Y, Cozzens K et al. Percutaneous Coronary Intervention for ST-Elevation Myocardial Infarction Before and During COVID in New York. *Am J Cardiol* 2021;142:25-34.10.1016/j.amjcard.2020.11.033

63. Hauguel-Moreau M, Pillière R, Prati G et al. Impact of Coronavirus Disease 2019 outbreak on acute coronary syndrome admissions: four weeks to reverse the trend. *J Thromb Thrombolysis* 2020;:1-2. https://www.ncbi.nlm.nih.gov/pmc/articles/PMC7323878/ 10.1007/s11239-020-02201-9

64. Kobo O, Efraim R, Saada M et al. The impact of lockdown enforcement during the SARSCoV-2 pandemic on the timing of presentation and early outcomes of patients with ST-elevation myocardial infarction. *PLoS One* 2020;15:e0241149.10.1371/journal.pone.0241149

65. Kwok CS, Gale CP, Kinnaird T et al. Impact of COVID-19 on percutaneous coronary intervention for ST-elevation myocardial infarction. *Heart* 2020;106:1805-1811.10.1136/heartjnl-2020-317650

66. Little CD, Kotecha T, Candilio L et al. COVID-19 pandemic and STEMI: pathway activation and outcomes from the pan-London heart attack group. *Open Heart* 2020;710.1136/openhrt-2020-001432

67. Nader J, Anselmi A, Tomasi J et al. Adult cardiac surgery during COVID-19 lockdown: Impact on activity and outcomes in a high-volume centre. *Arch Cardiovasc Dis* 2021;114:364-370.10.1016/j.acvd.2020.12.003

68. Pascual Calleja I, Álvarez Velasco R, Almendarez Lacayo M, Arboine Aguirre L, Avanzas Fernández P, Moris de la Tassa, César. Impact of the COVID-19 pandemic on acute myocardial infarction care times. *Emergencias* 2020;32:440-442. 43

69. Popovic B, Varlot J, Metzdorf PA, Jeulin H, Goehringer F, Camenzind E. Changes in characteristics and management among patients with ST-elevation myocardial infarction due to COVID-19 infection. *Catheter Cardiovasc Interv* 2021;97:E319-E326.10.1002/ccd.29114

70. Rodríguez-Leor O, Cid-Álvarez B, Pérez de Prado A et al. Impact of COVID-19 on ST-segment elevation myocardial infarction care. The Spanish experience. *Rev Esp Cardiol (Engl Ed)* 2020;73:994-1002.10.1016/j.rec.2020.08.002

71. Scholz KH, Lengenfelder B, Thilo C et al. Impact of COVID-19 outbreak on regional STEMI care in Germany. *Clin Res Cardiol* 2020;109:1511-1521.10.1007/s00392-020-01703-z

72. Garcia S, Stanberry L, Schmidt C et al. Impact of COVID-19 pandemic on STEMI care: An expanded analysis from the United States. *Catheter Cardiovasc Interv* 2021;98:217-222.10.1002/ccd.29154

73. Nappi C, Megna R, Acampa W et al. Effects of the COVID-19 pandemic on myocardial perfusion imaging for ischemic heart disease. *Eur J Nucl Med Mol Imaging* 2020;:1-7. https://www.ncbi.nlm.nih.gov/pmc/articles/PMC7417201/ 10.1007/s00259-020-04994-6

74. Quadri G, Rognoni A, Cerrato E et al. Catheterization laboratory activity before and during COVID-19 spread: A comparative analysis in Piedmont, Italy, by the Italian Society of Interventional Cardiology (GISE). *Int J Cardiol* 2021;323:288-291.10.1016/j.ijcard.2020.08.072

75. Casey L, Khan N, Healy DG. The impact of the COVID-19 pandemic on cardiac surgery and transplant services in Ireland's National Centre. *Ir J Med Sci* 2020;:1-5. https://www.ncbi.nlm.nih.gov/pmc/articles/PMC7335226/ 10.1007/s11845-020-02292-6

76. Elliott JM, Crozier IG. Decreases in cardiac catheter laboratory workload during the COVID-19 level 4 lockdown in New Zealand. *Intern Med J* 2020;50:1000-1003.10.1111/imj.14922

77. Iafrancesco M, Farina P, Bruno P et al. Impact of Covid-19 Pandemia on Provision of Surgical Treatment for Patients With Cardiovascular Disease. *Circulation* 2020;142:A15347-A15347. https://www.ahajournals.org/doi/abs/10.1161/circ.142.suppl_3.15347 10.1161/circ.142.suppl_3.15347

78. Ishii H, Amano T, Yamaji K, Kohsaka S, Yokoi H, Ikari Y. Implementation of Percutaneous Coronary Intervention During the COVID-19 Pandemic in Japan - Nationwide Survey Report of the Japanese Association of Cardiovascular Intervention and Therapeutics for Cardiovascular Disease. *Circ J* 2020;84:2185-2189.10.1253/circj.CJ-20-0708

79. Kwok CS, Gale CP, Curzen N et al. Impact of the COVID-19 Pandemic on Percutaneous Coronary Intervention in England: Insights From the British Cardiovascular Intervention Society PCI Database Cohort. *Circ Cardiovasc Interv* 2020;13:e009654.10.1161/CIRCINTERVENTIONS.120.009654

80. Mohamed MO, Kinnaird T, Curzen N et al. In-Hospital and 30-Day Mortality After Percutaneous Coronary Intervention in England in the Pre-COVID and COVID Eras. *J Invasive Cardiol* 2021;33:E206-E219. 44

81. Rodríguez-Caulo EA, Carnero Alcázar M, Garrido Jiménez JM, Barquero Aroca JM. National survey: Impact of COVID-19 on cardiovascular surgery services in Spain (SECCE-COVID19 Study. *Cirugía Cardiovascular* 2021;28:67-70. https://www.sciencedirect.com/science/article/pii/S1134009621000164 10.1016/j.circv.2021.01.003
82. Soylu K, Coksevim M, Yanık A, Bugra Cerik I, Aksan G. Effect of Covid-19 pandemic process on STEMI patients timeline. *Int J Clin Pract* 2021;75:e14005.10.1111/ijcp.14005
83. Rebollal-Leal F, Aldama-López G, Flores-Ríos X et al. Impact of COVID-19 outbreak and public lockdown on ST-segment elevation myocardial infarction care in Spain. *Cardiol J* 2020;27:425-426.10.5603/CJ.a2020.0098
84. Einstein AJ, Shaw LJ, Hirschfeld C et al. International Impact of COVID-19 on the Diagnosis of Heart Disease. *J Am Coll Cardiol* 2021;77:173-185.10.1016/j.jacc.2020.10.054
85. European Society of Hypertension Corona-virus Disease 19 Task Force. The corona-virus disease 2019 pandemic compromised routine care for hypertension: a survey conducted among excellence centers of the European Society of Hypertension. *J Hypertens* 2021;39:190-195.10.1097/HJH.0000000000002703
86. Sankaranarayanan R, Hartshorne-Evans N, Redmond-Lyon S et al. The impact of COVID-19 on the management of heart failure: a United Kingdom patient questionnaire study. *ESC Heart Fail* 2021;8:1324-1332.10.1002/ehf2.13209
87. Wosik J, Clowse MEB, Overton R et al. Impact of the COVID-19 pandemic on patterns of outpatient cardiovascular care. *Am Heart J* 2021;231:1-5.10.1016/j.ahj.2020.10.074
88. Chagué F, Boulin M, Eicher J et al. Impact of lockdown on patients with congestive heart failure during the coronavirus disease 2019 pandemic. *ESC Heart Fail* 2020;10.1002/ehf2.13016
89. Wadhera RK, Shen C, Gondi S, Chen S, Kazi DS, Yeh RW. Cardiovascular Deaths During the COVID-19 Pandemic in the United States. *J Am Coll Cardiol* 2021;77:159-169.10.1016/j.jacc.2020.10.055
